# A state-of-the-art review of diffusion model applications for microscopic image and micro-alike image analysis

**DOI:** 10.3389/fmed.2025.1551894

**Published:** 2025-07-16

**Authors:** Yan Liu, Tao Jiang, Rui Li, Lingling Yuan, Marcin Grzegorzek, Chen Li, Xiaoyan Li

**Affiliations:** ^1^College of Medicine and Biological Informaton Engineering, Northeastern University, Shenyang, China; ^2^College of Intelligent Medicine, Chengdu University of Traditional Chinese Medicine, Chengdu, China; ^3^University of Lübeck, Lübeck, Germany; ^4^Histopathology Department, Liaoning Cancer Hospital, Shenyang, China

**Keywords:** microscopic image, micro-alike image, diffusion model, image generation, image segmentation, image analysis

## Abstract

Diffusion models, a class of deep learning models based on probabilistic generative processes, progressively transform data into noise and then reconstruct the original data through an inverse process. Recently, diffusion models have gained attention in microscopic image analysis for their ability to process complex data, extract valuable information, and enhance image quality. This review provides an overview of diffusion models in microscopic images and micro-alike images, focusing on three commonly used models: DDPM, DDIM, and SDEs. We explore their applications in image generation, segmentation, denoising, classification, reconstruction and super-resolution. It shows their notable advantages, particularly in image generation and segmentation. Through simulating the imaging process of biological samples under the microscope, diffusion model can generate high-quality synthetic microscopic images. The generated images serve as a powerful tool for data augmentation when training deep learning models. Diffusion model also excels in microscopic image segmentation. It enables to accurately segment different cellular regions and tissue structures by simulating the interactions between pixels in an image. The review includes 31 papers, with 13 on image generation, nine on segmentation, and the remainder on other applications. We also discuss the strengths, limitations, and future directions for diffusion models in biomedical image processing.

## 1 Introduction

### 1.1 microscopic images and micro-alike images

Micrographs are images captured using microscopes or other high-resolution imaging devices to observe and study tiny structures in the microscopic world. These images typically provide magnified views of biological, material, or other microscopic samples and are indispensable tools in cell biology. Micrographs provide scientists with valuable insights into the structure and function of cells, making them crucial not only in the medical field but also in environmental science and materials chemistry research. On the other hand, micro-alike images, typically obtained through other high-resolution imaging techniques or devices, share similar properties with conventional microscopic images, such as high resolution and the ability to observe and analyze minute structures. Consequently, micro-alike images also hold comparable research value and are applicable in similar scenarios as microscopic images.

**Figure 1:**
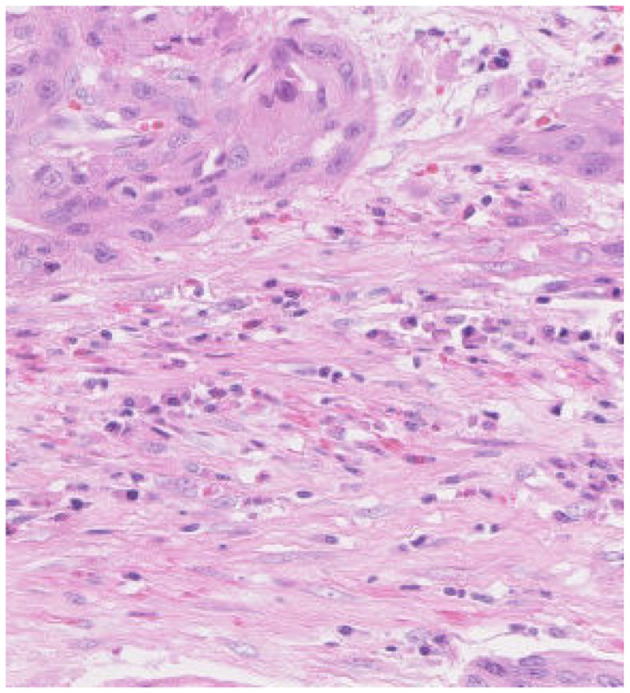
Histopathologic image of intestines tissue sections observed under the microscope.

**Figure 2:**
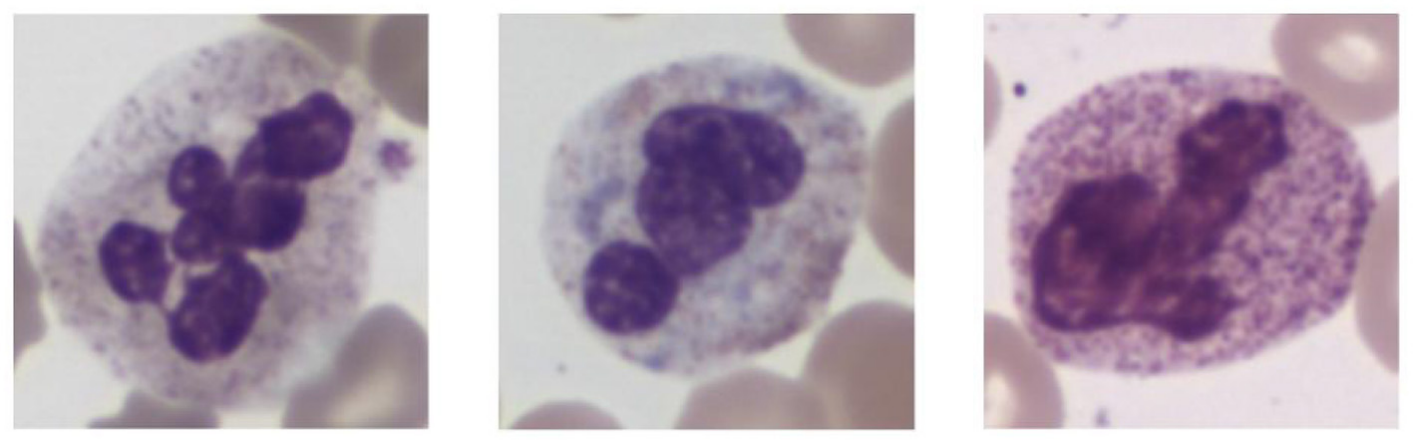
Examples of cellular images displaying various abnormalities. From left to right these are hypersegmentation, D^..^ohle bodies and hypergranulation of neutrophil. Reproduced with permission from “Examples of images of cells with different abnormalities” by Louise Zettergren and Fanny Nilsson, licensed under CC BY 4.0.

In histopathological studies, microscopic images play a crucial role in medical research, diagnosis, and treatment. In pathology, microscopic images of tissue sections offer detailed views of the internal structure and lesions of biological tissues. An example of a histopathological image is shown in Figure 1. These images are critical for disease diagnosis, pathology research, and decision making in medical practice. It can help doctors and researchers better understand and diagnose diseases (1). Ajay proposed a method to identify the extent of lymphocyte infiltration in histopathological images of breast cancer, offering a new quantitative approach for pathological assessment (2). Additionally, cellular images have wide-ranging applications in pathology. In clinical trials, cytological examination can detect abnormal cell morphology, including changes in cell size, nuclear morphology, and organelle structure, aiding in the early detection of lesions. For example, neutrophils with abnormal features are shown in Figure 2. These are hypersegmentation, D^..^ohle bodies and hypergranulation of neutrophil. It is usually used to diagnose a number of malignancies and leukaemias in the hematological system (3). Finally, micrographic techniques can be combined with methods such as fluorescent labeling. Thus, the localisation and distribution of biomolecules (e.g. proteins, nucleic acids, etc.) in cells and tissues can be observed and analyzed (4). In environmental science research, microscopic image can be used to observe microbial community structure, abundance and distribution. Through micrographic techniques to study the relationships of microbial diversity, ecological functions with environmental change. It provides effective technical means and important data support for the study of environmental microbial ecology. For example, algae are good bioindicators for water pollution assessment. Using micrographic techniques it is possible to measure changes in algal species and abundance to identify changes in water quality and nutrient status (5).

**Figure 3:**
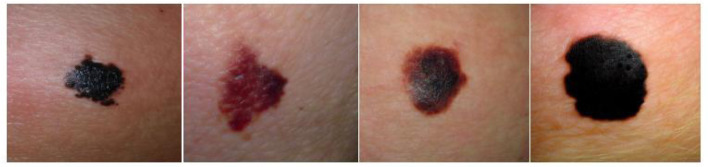
Representative images of various melanoma, highlighting differences in lesion appearance, including variations in type, size, color, and shape. Reproduced with permission from “Synthetic melanoma images generated by the stable diffusion model after fine-tuning it with melanoma images using the input text prompt “melanoma””, by Mohamed Akrout, Bálint Gyepesi, Péter Holló, Adrienn Poór, Blága Kincsõ, Stephen Solis, Katrina Cirone, Jeremy Kawahara, Dekker Slade, Latif Abid, Máté Kovács and István Fazekas, licensed under CC BY 4.0.

Micro-alike images, such as skin cancer and fundus images, are typically captured using specialized imaging equipment. For example, skin cancer images, often obtained with devices like dermoscopy (6), are vital in dermatological medicine, allowing for the assessment of lesion type, size, color, shape, and other characteristics. These images aid in diagnosing and monitoring skin cancer and other skin conditions, as well as determining the malignancy and depth of lesions (7). Figure 3 shows several common melanoma images. Similarly, fundus images, captured using specialized fundus cameras, are designed to observe and analyze the posterior structures of the eye, including the retina, optic nerve, blood vessels, macula, and optic disc.

**Figure 4:**
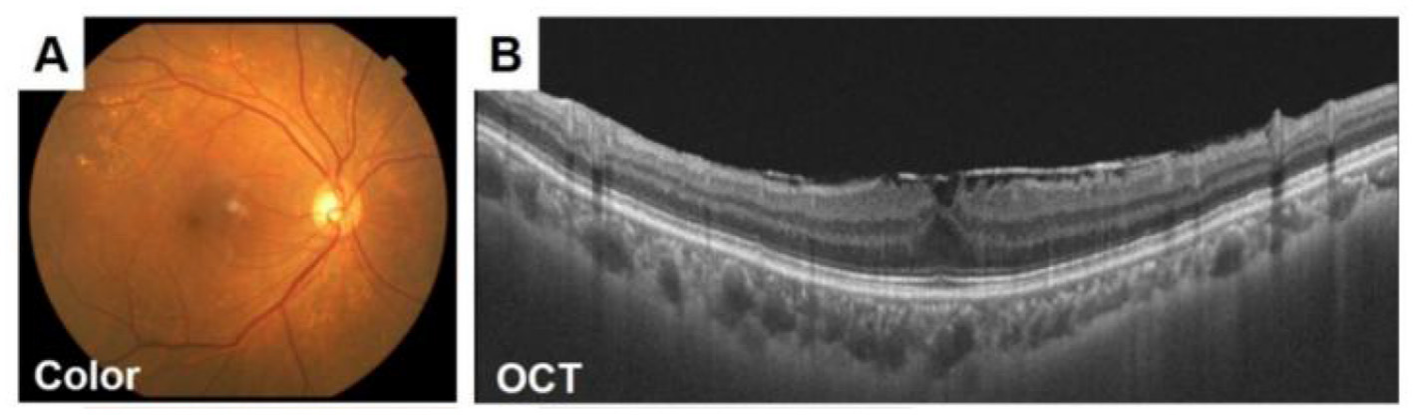
Fundus images of a 70-year-old woman with an ERM. **(A)** The retinal nerve fiber layer (RNFL) defect is difficult to detect in the ocular fundus image obtained by a conventional fundus camera **(A)**. **(B)**Epiretinal membrane can be seen in OCT images. Adapted with permission from “Fundus images of a 70-year-old woman with an ERM” by Hiroto Terasaki, Shozo Sonoda, Masatoshi Tomita and Taiji Sakamoto, licensed under CC BY 4.0.

An image obtained by optical coherence tomography (OCT) (8) is shown in Figure 4. These images are crucial tools for doctors to diagnose and monitor eye health (9). Studies have shown that fundus imaging is associated with a wide range of diseases. For example, Liesenfeld et al. found that regular digital fundus imaging allows for the early detection and treatment of diabetic retinopathy, which is common among diabetics (10). Parham proposed a CNN-based method for retinal analysis, enabling the automated identification of lesions such as exudates, hemorrhages, and microaneurysms in fundus images (11).

Endoscopic images are another type of micro-alike image. With technological advancements, endoscopic imaging devices have evolved from magnifying endoscopes and autofluorescence imaging (AFI) to confocal laser microendoscopy. These high-resolution endoscopic images, which can reach up to 1 million pixels, provide endoscopists with clearer views of capillaries and submucosal vessels (12).

### 1.2 Diffusion model

In recent years, deep generative modeling has gained significant attention, with three mainstream approaches emerging: variational autoencoders (VAEs) (13), generative adversarial networks (GANs) (14), and diffusion models (15). GANs face challenges such as unstable training, which can lead to model crashes and a lack of diversity in generated samples (16). VAEs, on the other hand, often produce more blurred images because their training objective is to maximize data likelihood (17). In contrast, diffusion models have shown significant potential due to their relatively stable training process and robustness to noise. These models operate through two key steps: a forward diffusion process, where noise is added to corrupt the training data into pure Gaussian noise, and a reverse denoising process, where the noise is gradually removed to restore the original data structure (18). As cutting-edge generative models, diffusion models are being extensively researched for their applications across various fields.

To date, diffusion models have been utilized in a wide range of generative modeling tasks. Following the advancements of VAEs and GANs, diffusion models have made significant progress in fields such as computer vision and medicine. Their wide applicability extends to micrography, where they are not only used for image generation but also for image segmentation, denoising, and various image restoration tasks, including super-resolution and translation (19).

Image generation (20, 21): Image Generation is the process of automatically generating images using algorithms and deep learning techniques. This technique usually relies on neural network models, especially Generative Adversarial Networks, Variational Auto-Encoders and Diffusion Models. These models are can generate new images from random noise, pre-existing images, or conditional information such as text descriptions. Generating synthetic images through diffusion models can be used for data augmentation, effectively addressing data scarcity and reducing the risk of model overfitting. A novel generative framework (22) combines the diffusion process with composable modules, allowing dynamic combination of image parts, thus enhancing flexibility in generating complex images with better semantic information and structure.

Image classification (23): Microscopic image classification is an important task in computer vision, especially in the fields of medical image analysis, pathology, and cell biology. The aim is to classify microscopic images into different categories based on their content (such as pathological state). However, Medical data often face issues of imbalance, especially with rare diseases or abnormal cells. Diffusion models can generate high-quality, photorealistic images to enhance image classification performance by applying both generated and original images to classifiers (24, 25).

Image denoising: Microscopic images are always affected by different types of noise. The development of denoising techniques is crucial for image clarity and analysis. Image Denoising improves the quality of the image, which in turn improves the accuracy of subsequent analysis. Diffusion models provide adaptive denoising based on image characteristics and noise type, preserving structure and edge information while avoiding excessive smoothing or blurring, ensuring clarity and recognizability in the denoised image (26).

Super-resolution reconstruction (27): Super-Resolution Reconstruction is a technique for recovering a high-resolution (HR) image from a low-resolution (LR) image. The goal is to enhance the resolution and detail of the image, making it clearer for more accurate analysis and processing. Diffusion models can enhance image quality and details through the diffusion process. The Super-Resolution via Repeated Refinement (SR3) framework, based on DDPM, progressively improves low-resolution images via iterative denoising to produce high-resolution outputs (28).

Image segmentation (29, 30): Microscopic image segmentation is one of the key tasks in microscopic image analysis. It is mainly used to extract regions of interest (cells, tissues, subcellular structures, etc.) from images acquired under a microscope. With precise segmentation, researchers are able to perform more deep analysis such as cell counting, tissue analysis, and lesion detection. Traditional segmentation methods require significant time and cost for labeling training data. Diffusion models, however, learn similarities and correlations between samples, propagating labeling information from a small number of labeled samples to unlabeled ones, thereby reducing reliance on labeled data and achieving comparable or better performance than traditional methods (31).

Image-to-image translation (32, 33): Diffusion models are also effective in image-to-image translation, converting images from one domain to another with different visual characteristics and semantic meanings (34). Their adaptive nature allows for efficient conversion by adjusting diffusion process parameters based on input image (35).

### 1.3 Motivation of this review

Microscopic and micro-alike images play a critical role in various fields. By extracting features from these images, a wide range of image analysis algorithms can be applied to achieve different objectives. For instance, in pathology, microscopic image analysis is essential for tumor diagnosis, histopathological analysis, and cancer detection. Medical professionals can determine the type, extent, and treatment options for diseases by examining tissue sections (36). In biological research, microscopic images are used to study cell structure, function, and biomolecular interactions, helping biologists understand biological processes and mechanisms within organisms (37).

However, microscopic images often have complex structures and rich details that traditional analysis methods struggle to process effectively. Traditional techniques tend to extract only basic features, making accurate analysis and identification of complex structures and cellular morphology difficult and time-consuming (38). In contrast, diffusion models, as non-linear models, are well-suited to handle these complexities. They capture higher-order features and non-linear relationships, enhancing the expressive and fitting capabilities of the models (18).

Over the past two years, there has been a growing body of work exploring the application of diffusion modeling in various domains, particularly in computer vision and medical imaging. For example, Croitoru et al. (39) outlines three subclasses of diffusion models–DDPM, noise-conditioned score networks (NCSNs) (40), and Stochastic Differential Equations (SDEs) (41)–which have shown superior results in tasks such as image generation, segmentation, and image-to-image translation. However, only two of the 114 applications discussed in this paper focus on microscopic image analysis. The work of Kazerouni et al. (42) systematically reviews advances in diffusion models within medical image analysis, including tasks like image-to-image conversion, reconstruction, alignment, classification, segmentation, and denoising. Of the 192 papers cited, only six deal with microscopic images.

In Zhang et al. (43), the current state of diffusion modeling for text-guided image generation is reviewed. This paper cites 150 references, all focused on applications in computer vision and natural images, and further examines methods for guiding image generation at different diffusion prior positions. The work ofLi et al. (44) addresses the challenges of using diffusion models in image processing, particularly in non-autoregressive (NAR) text generation. While NAR methods reduce computational time, they significantly decrease the accuracy of generated images. This review focuses on the contributions of NAR text generation in natural images and does not cover microscopic images. In Cao et al. (45), new conditioning techniques in text-guided image generation are discussed, including condition-specific, multi-conditional, and universal controllable generation, with a primary focus on applications involving natural images.

The study by (19) explores ways to enhance diffusion model performance, focusing on three key areas: efficient sampling, improved likelihood estimation, and handling specially structured data. The paper also proposes combining diffusion models with other generative models, such as VAEs, GANs, and Energy-based Models, to broaden their applicability. Despite citing 349 references, none specifically address microscopy images. The work of Chen et al. (46) provides a comprehensive survey of diffusion model advancements in fields such as computer vision, audio, medicine, bioinformatics, and others. It covers theoretical advances in both unconditional and conditional diffusion models and discusses optimization techniques, including black-box optimization. Out of 216 references, only one pertains to microscopic images. Similarly, Cao et al. (15) investigates different applications of diffusion models across computer vision, natural language processing, and medicine, focusing on optimized diffusion models with techniques like sample acceleration, diffusion process design, and ELBO optimization. Out of 92 references on image analysis, only two relate to microscopic images.

The review in Li et al. (47) systematically outlines the use of diffusion models in image restoration, discussing two main approaches: supervised diffusion-based models and zero-bounce diffusion-based models. It summarizes 64 papers, covering natural images, medical images, and biomolecules. Guo et al. (48) summarized the application of diffusion models to biomolecules, highlighting recent advances in protein molecule design, small molecule design, cryo-electron microscopy image analysis, and single-cell data analysis. Of the 273 articles cited, only two were related to micrograph studies.

Table 1 illustrates the number of papers related to microscopic and micro-alike images within the cited literature of the reviewed works. An analysis of this figure and the related literature reveals that while diffusion models are gaining attention, there is still limited research specifically focused on microscopic and micro-alike image analysis. Most studies continue to concentrate on natural image analysis in computer vision, protein molecular design in bioinformatics, and related areas.

**Table 1:** A summary and comparison of the primary surveys in the field of diffusion model, where percentage is the ratio of the third column to the second column.

**Artical**	**Number of references cited in the review**	**Number of references related to microscopic and micro-alike image**	**Percentage**	**Field of application**
Diffusion models in vision: A survey (39)	114	2	1.75%	This paper comprehensive review of articles on diffusion models applied in vision.
A survey on generative diffusion models (15)	92	2	2.17%	This paper studies different applications of diffusion model in different fields about computer vision, natural language processing, medicine
Diffusion models in medical imaging: A comprehensive survey (42)	192	6	3.12%	This paper summaries diffusion models applied in medical images. However, it focuses on mainly in CT and MRI.
Diffusion models: A comprehensive survey of methods and applications (19)	349	0	0	This paper concentrates on the application of diffusion model to image synthesis, video generation, and molecular design.
Text-to-image diffusion model in generative ai: A survey (43)	150	0	0	This paper presents a review of state-of-the-art methods on text-conditioned image synthesis, i.e. text-to-image.
Diffusion models for image restoration and enhancement-a comprehensive survey (47)	64	0	0	This paper provides an overview of diffusion model for image restoration on natural images, medical images, and biomolecules.
Diffusion models for non-autoregressive text generation: A survey (44)	53	0	0	This paper summarizes diffusion Modeling for Non-Autoregressive Text Generation in natural language field.
Controllable generation with text-to-image diffusion models (45)	249	0	0	This paper presents improved diffusion model for text-conditioned image synthesis in natural language field.
Diffusion models in bioinformatics and computational biology (48)	273	2	0.73%	This paper primarily summarizes the application of diffusion models in bioinformatics.
An overview of diffusion models: Applications, guided generation, statistical rates and optimization (46)	216	1	0.46%	This paper concludes advances of diffusion models in the fields of computer vision, audio, reinforcement learning, and computational biology.
A state-of-the-art review of diffusion model applications for microscopic image and micro-alike image analysis	119	32	26.89%	Our paper concludes three commonly used models: DDPM, DDIM, and SDEs. We explore their applications in image generation, segmentation, classification, denoising, reconstruction and super-resolution.

### 1.4 Structure of the review

This review provides an overview of the application of diffusion models to microscopic and micro-alike images. In Section 2, we introduce the basic theory of diffusion models, focusing on the three primary approaches: NCSN, DDPM, and SDE. Section 3 delves into advances in diffusion models for image generation, particularly conditionally guided generation, and discusses their benefits for medical research and education. Sections 4 and 5 summarize the applications of diffusion models in segmentation and other tasks, providing a detailed overview of different use cases. In Section 6, we evaluate the strengths, weaknesses, and areas for improvement of diffusion models, as discussed in the previous sections. Finally, Section 7 offers conclusions and explores potential future research directions in diffusion models.

**Figure 5:**
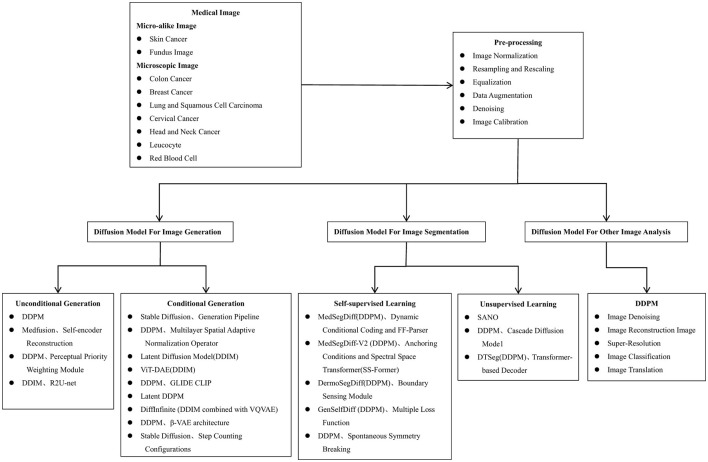
The algorithmic process of using diffusion models for microscopic image analysis, encompassing stages such as image acquisition, pre-processing, image generation, segmentation, and other image analysis methods.

**Figure 6:**
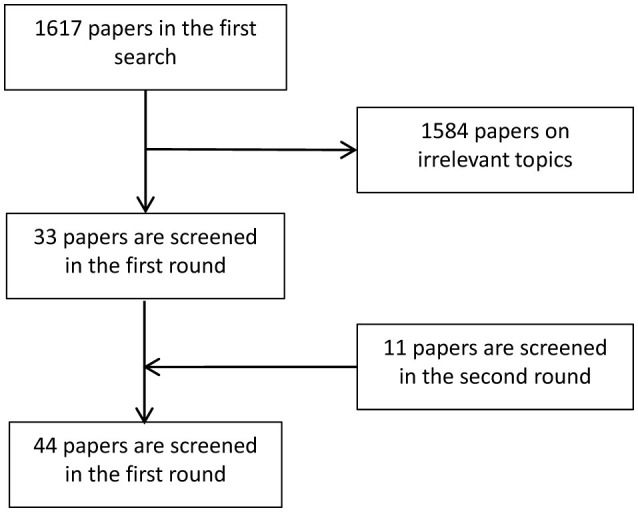
Flow chart illustrating the screening process for selecting relevant papers.

Figure 5 summarizes the general flow of microscopic image analysis using diffusion models over recent years, highlighting popular methods for each type of analysis. Despite the growing interest, there is a scarcity of literature specifically addressing the application of diffusion models to microscopic and micro-alike images. Figure 6 shows the whole process of filtering articles from keywords such as histopathological images, cellular images, and microscopic images through Google Scholar.

On the one hand, the relevant papers are filtered through the references of the related reviews. On the other hand, relevant papers are searched by keywords of diffusion model, histopathological images, microscopic images and other keywords in Google Scholar. The literature is filtered according to its content. Part of the literature is the application of diffusion model on CT images, MRI, which does not involve microscopic images and then are excluded. A total of 44 relevant papers are screened from 1,717 articles on both occasions.

In summary, this review is the first to provide a comprehensive overview of the application of diffusion models to microscopic and micro-alike images, covering a wide range of tasks including image generation, segmentation, classification, denoising, image reconstruction, and super-resolution.

## 2 Basic knowledge of diffusion model

In this section, we outline two fundamental formulations of diffusion models: Denoising Diffusion Probabilistic Models (DDPMs) and Stochastic Differential Equations (SDEs). We describe the process of adding noise and the methods for generating new data in the reverse process for both formulations. Additionally, to accelerate the sampling process in DDPMs, we introduce a new generative model called the Denoising Diffusion Implicit Model (DDIM). This model achieves more efficient sample generation by employing a non-Markovian diffusion process, resulting in faster generation, improved sample quality, and support for semantic interpolation in latent spaces.

### 2.1 Denoising Diffusion Probabilistic Models (DDPMs)

DDPM is a probabilistic model designed for image denoising (18, 49), which characterizes the relationship between noise and the signal in an image. It removes noise by controlling the diffusion process to recover a clear image, utilizing parameter estimation and image denoising by maximizing the log-likelihood between observations and model predictions.

#### 2.1.1 Forward process

**Figure 7:**
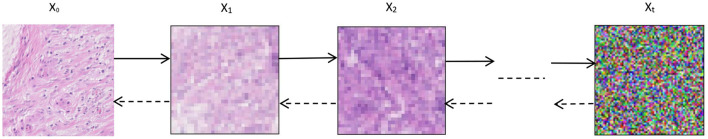
Denoising Diffusion Probabilistic Models. *x*_0_ → *x*_*t*_ is the forward process of DDPM, *x*_*t*_ → *x*_0_ is the reverse process of DDPM.

Given an initial data distribution *x*_0_ ~ *q*(*x*), Gaussian noise can be continuously added to the distribution(The standard deviation of Gaussian noise is determined from a fixed value of β_*t*_). The mean value is determined from a fixed value β_*t*_ and the state *x*_*T*_ at the current moment *t*. As the time step *t* increases (*t*→*T*) the final data *x*_*T*_ becomes an individual Gaussian distribution, as shown in Figure 7. The initial moment *q*(*x*_0_) is the distribution of the real image. We can do this by randomly sampling an image from the real image in the training datas, denoted as *x*~ *q*(*x*_0_). Then the forward process *q*(*x*_*t*_|*x*_*t*−1_) as shown in Figure 7 means that adding Gaussian noise to the image *x*_*t*−1_ to get *x*_*t*_ at each step of the forward. The mean of the added Gaussian noise is $\mu_{t}=\sqrt{1-\beta_{t}}x_{t-1}$ and the variance is σ_*t*_ = β_*t*_*I*:


(1) $$\begin{array}{l} q\left(x_{t}|x_{t-1}\right)=N\left(x_{t};\sqrt{1-\beta_{t}}x_{t-1},\beta_{t}\text{I}\right) \end{array}$$

The process of obtaining *x*_*t*_ from *x*_*t*−1_ satisfies the distribution of $N\left(x_{t};\sqrt{1-\beta_{t}}x_{t-1},\beta_{t}\text{I}\right)$. Thus we see that this noise is only determined by β_*t*_ and *x*_*t*−1_, which is a fixed value rather than a learnable process. Therefore, as long as we have *x*_0_ and determine in advance a fixed value for each step β_1_, ⋯ , β_*T*_, we can roll out the noise-added data *x*_1_, ⋯ , *x*_*T*_ for any step. According to the previous description, the forward diffusion process of DDPM is a Markov process. Then the posterior probability distribution from input *x*_0_ to *x*_*T*_ can be expressed as follow:


(2) $$\begin{array}{l} q\left(x_{1:T}|x_{0}\right)=\overset{T}{\underset{t=1}{\prod }}q\left(x_{t}|x_{t-1}\right) \end{array}$$

We let α_*t*_ = 1−β_*t*_,$\bar{\alpha_{t}}=\prod_{i=1}^{t}\alpha_{i}$, then there is β_*t*_ = 1−α_*t*_. Besides, via the original image *x*_0_ and β_*t*_, it is possible to sample *x*_*t*_ of any moment:


(3) $$\begin{array}{l} x_{t}\sim q\left(x_{t}|x_{0}\right)=N\left(x_{t};\sqrt{\bar{\alpha_{t}}}x_{0},\left(1-\bar{\alpha_{t}}\right)\text{I}\right) \end{array}$$

#### 2.1.2 Reverse process

The forward process of DDPM is to continuously add noise to the known data. The implicit variable *x*_*T*_ can be considered as an isotropic Gaussian distribution when the time step *T* → ∞. While the reverse process *p*(*x*_*t*−1_|*x*_*t*_) of DDPM is a denoising process. In other words, we first take a random sample of a 2D Gaussian noise at the time t and then progressively denoise it. The final result is a generated image that is consistent with the distribution of the real image *x*_0_.

The core process of DDPM is how the above denoising process is performed. Since the reverse diffusion process is unknown. We can learn this denoising process using a neural network. In the process of diffusion, the distribution *x*_*t*_ of moments t is known. Thus the purpose of the constructed neural network is to learn the probability distribution function of *x*_*t*−1_ based on *x*_*t*_. In summary, the reverse process of DDPM can be modeled as *p*(*x*_*t*−1_|*x*_*t*_). At each moment in the forward diffusion process adds Gaussian noise to the implicit variables. Then the reverse denoising process filters out Gaussian noise as well. Theoretically, a random Gaussian noise is determined by the parameter mean μ_θ_ and variance Ω_θ_. So, *p*(*x*_*t*−1_|*x*_*t*_) can be defined as:


(4) $$\begin{array}{l} p_{\theta }\left(x_{t-1}|x_{t}\right)=N\left(x_{t-1};\mu_{\theta }\left(x_{t},t\right),\Sigma_{\theta }\left(x_{t},t\right)\right) \end{array}$$

The inverse process of DDPM is also a Markov process. The inverse process of the diffusion model is obtained via Markov chain defined as:


(5) $$\begin{array}{l} p_{\theta }\left(x_{0:T}\right)=p\left(x_{T}\right)\cdot \overset{T}{\underset{t=1}{\prod }}p_{\theta }\left(x_{t-1}|x_{t}\right)) \end{array}$$

where *p*(*x*_*T*_) = *N*(*x*_*t*_; 0, **I**) is a randomly sampled Gaussian noise; *p*_θ_(*x*_*t*−1_|*x*_*t*_) denoted the Gaussian distribution for which the mean and variance need to be calculated.

#### 2.1.3 Training losses

The loss function of the DDPM is based on the negative likelihood logarithm plus a KL dispersion. Thus, forming an upper bound on the negative likelihood logarithm.


(6) $$\begin{array}{r} -\text{log}p_{\theta }\left(x_{0}\right)\leq -\text{log}p_{\theta }\left(x_{0}\right)+D_{\text{KL}}\left[q\left(x_{1:T}|x_{0}\right)||p_{\theta }\left(x_{1:T}|x_{0}\right)\right]\\ =E_{x\sim q\left(x_{1:T}|x_{0}\right)}\left[\text{log}\frac{q\left(x_{1:T}|x_{0}\right)}{p_{\theta }\left(x_{0:T}\right)}\right]\\ =L_{\text{LVB}} \end{array}$$

As every time state variable of the diffusion process satisfies the Markov distribution, so the above equation can be written as:


(7) $$\begin{array}{r} L_{\text{LVB}}=-\underset{L_{\text{Res}}}{\underset{︸}{E_{x\sim q\left(x_{1}|x_{0}\right)}\left[\text{log}p_{\theta }\left(x_{0}|x_{1}\right)\right]}}+\underset{L_{T}}{\underset{︸}{D_{\text{KL}}\left(q\left(x_{T}|x_{0}\right)||p_{\theta }\left(x_{T}\right)\right)}}+\\ \Sigma_{t=2}^{T}\underset{L_{t-1}}{\underset{︸}{E_{x\sim q\left(x_{t}|x_{0}\right)}\left[D_{\text{KL}}\left(q\left(x_{t-1}|x_{t},x_{0}\right)||p_{\theta }\left(x_{t-1}|x_{t}\right)\right)\right]}} \end{array}$$

where *L*_RES_ denotes the image reconstruction loss function. It serves to reconstruct the original data and optimizes a negative log-likelihood estimate. *L*_*T*_ denotes a priori information match, which computes the final noise input using KL Divergence. The denoising loss function *L*_*t*−1_ calculates the KL Divergence between the true posterior distribution *q*(*x*_*t*−1_|*x*_*t*_, *x*_0_) and the predicted distribution *p*_θ_(*x*_*t*−1_|*x*_*t*_). Since the goal of DDPM is to make the real denoising process as consistent as possible with the model predicted denoising process. Simplifying *L*_*t*−1_ by means of reparameterisation, the final loss function of the DDPM simplifies to:


(8) $$\begin{array}{l} L_{\text{simple}-\text{DDPM}}=E_{x,t,\epsilon }||\epsilon_{t}-{\hat{\epsilon }}_{\theta }\left(\sqrt{\alpha_{t}}x_{0}+\sqrt{1-\bar{\alpha_{t}}}\epsilon ,t\right)|{|}_{2}^{2} \end{array}$$

### 2.2 Stochastic Differential Equations (SDEs)

SDEs are a class of mathematical modeling methods that use stochastic differential equations to describe the evolution of a system. In the field of image processing, SDEs are commonly used to model the evolution of images. These methods work on the basis that the image is a random process.

The forward diffusion of SDEs can be denoted in terms of both drift and random noise components:


(9) $$\begin{array}{l} dx=f\left(x,t\right)dt+g\left(t\right)d\omega \end{array}$$

where f is a vector function referred to as the drift coefficient. *g*(*t*) is a real function denoted as the diffusion coefficient. ω denotes standard Brownian motion and dω is infinitesimal white noise. The solution of the SDE is a continuous collection *x*(*t*)_0:*T*_ of random variables. These random variables track the random trajectory of time index *t* from 0 to *T*. Denote the marginal probability density function of *x*(*t*) by *p*_*t*_(*x*). At *t*=0, *p*_0_(*x*) = *p*(*x*). No noise is mixed into the original data distribution at the initial moment. After a sufficiently long period of time *T*, with the mixing of noise of increasing size, *p*(*x*) becomes a tractable noise distribution (e.g., Gaussian), denoted as π(*x*), which is known as the prior distribution. *p*_*T*_(*x*) corresponds to the case of maximum noise for limited noise layers.

In the case of a limited number of noise sizes (DDPMs), we generate samples by gradually reducing the noise through a reverse process. Similarly, we use an inverse SDE to reverse the noise mixing process for sample generation for an infinite number of noise layers. The SDE form of the inverse diffusion process is as‘ follows:


(10) $$\begin{array}{l} dx=\left[f\left(x,t\right)-g^{2}\left(t\right){▽}_{x}\text{log}p_{t}\left(x\right)\right]dt+g\left(t\right)d\omega \end{array}$$

where *dt* is an infinitesimal time step, this SDE needs to be solved inversely, from *t*=*T* to *t*=0. ▽_*x*_*logp*_*t*_(*x*) is the score function of *p*_*t*_(*x*) and log is the gradient of the data distribution. In order to estimate the score function, we train a time-dependent score-based model *s*_θ_(*x, t*), making *s*_θ_(*x, t*) = ▽_*x*_*logp*_*t*_(*x*). In this way, an estimated inverse SDEs can then be obtained:


(11) $$\begin{array}{l} dx=\left[f\left(x,t\right)-g^{2}\left(t\right)s_{\theta }\left(x\right)\right]dt+g\left(t\right)d\omega \end{array}$$

Therefore, we can start from *x*(*T*) = π and obtain *x*(0) by solving the above reverse SDEs.

### 2.3 Denoising Diffusion Implicit Models(DDIM)

For diffusion model, the biggest drawback is that we need to set a long diffusion step to get good results, which leads to slower generation of samples. DDIM (50) and DDPM have the same training goals. However, it no longer restricts the diffusion process to be a Markov chain. This allows DDIM to use smaller sampling steps to speed up the generation process. Another feature of DDIM is that the process of generating samples from a random noise is a deterministic process.

Based on the above analysis, the inference distribution of DDIM is defined as:


(12) $$\begin{array}{l} q_{\sigma }\left(x_{1:T}|x_{0}\right)=q_{\sigma }\left(x_{T}|x_{0}\right)\overset{T}{\underset{t=2}{\prod }}q_{\sigma }\left(x_{t-1}|x_{t},x_{0}\right) \end{array}$$

**Figure 8:**
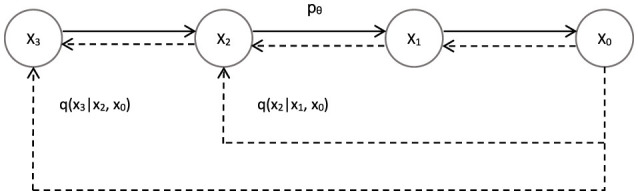
Skip-step sampling of DDIM: non-Markov chain. Breaking the Markov assumption of the model's original forward model, a specific backward model is found that makes that backward process deterministic.

Here it has to be satisfied *q*_σ_(*x*_*T*_|*x*_0_) = $N\left(\sqrt{\alpha_{T}}x_{0},\left(1-\alpha_{T}\right)\text{I}\right)$ and all of the *t*≥2 at the same time. Then there is:


(13) $$\begin{array}{l} q_{\sigma }\left(x_{t-1}|x_{t},x_{0}\right)=N\left(x_{t-1};\sqrt{\alpha_{t-1}}x_{0}+\sqrt{1-\alpha_{t-1}-\sigma_{t}^{2}}\frac{x_{t}-\sqrt{\alpha_{t}}x_{0}}{\sqrt{1-\alpha_{t}}},\sigma_{t}^{2}\text{I}\right) \end{array}$$

where the forward process is *q*_σ_(*x*_*t*_|*x*_*t*−1_, *x*_0_). Since the generation of *x*_*t*_ depends not only on *x*_*t*−1_ but also on *x*_0_. Hence, it's a non-Markov chain. As shown in the Figure 8:

Like DDPM, DDIM also uses neural networks ϵ_θ_ to predict noise. Then according to the form of *q*_σ_(*x*_*t*_|*x*_*t*−1_, *x*_0_), we can use the following formula to generate *x*_*t*−1_ from *xt* in the generation phase:


(14) $$\begin{array}{l} x_{t-1}=\sqrt{\alpha_{t-1}}\underset{predictedx_{0}}{\underset{︸}{\left(\frac{x_{t}-\sqrt{1-\alpha_{t}}\epsilon_{\theta }\left(x_{t},t\right)}{\sqrt{\alpha_{t}}}\right)}}+\underset{directionpointingtox_{t}}{\underset{︸}{\sqrt{1-\alpha_{t-1}-\sigma_{t}^{2}}\cdot \epsilon_{\theta }\left(x_{t},t\right)}}\\ \;+\underset{radonnoise}{\underset{︸}{\sigma_{t}\epsilon_{t}}} \end{array}$$

Where the generation process is divided into three parts: One is for the prediction of *x*_0_. The second is made up of the parts that point to *x*_*t*_. Third is random noise(ϵ_θ_is the noise independent of *x*_*t*_). Further define σ as follow:


(15) $$\begin{array}{l} \sigma_{t}^{2}=\eta \cdot \sqrt{(1-\alpha_{t-1}/\left(1-\alpha_{t}\right)}\sqrt{\left(1-\alpha_{t}/\alpha_{t-1}\right)} \end{array}$$

For Equation 15, consider two cases. When η = 1, at this point the forward process becomes a Markov chain and the generation process is the same as DDPM. The other case is η = 0, on this occasion the generation process is not subject to random noise. The model in this case is called DDIM. Once the initial random noise *x*_*T*_ is determined, then sample generation for DDIM becomes a deterministic process.

## 3 Application of image generation

**Figure 9:**
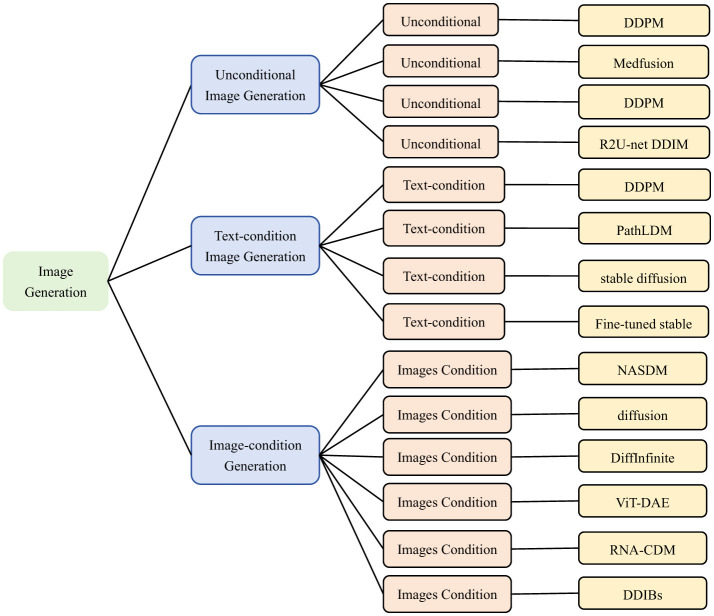
Classification of microscopic image generation based on diffusion model. Unconditional Image Generation: (3, 52, 55, 56). Text-condition Image Generation: (59, 62, 67, 70). Images-condition Image Generation: (24, 73, 78, 82, 85). We use the following abbreviations in the architecture column: Medfusion, Medical Image Fusion; PathLDM, Pathology Latent Diffusion Model; NASDM, Nuclei-Aware Semantic Diffusion Models; DiffInfinite(Diffusion-based Infinite Mask-Image Synthesis; ViT-DAE, Vision Transformer-driven Diffusion Autoencoder; RNA-CDM, RNA-Conditional Diffusion Mode; DDIBs, Dual Diffusion Implicit Bridges.

Micrographic image generation is a process of automatically generating micrographic images using deep learning and generative model. These images are usually of very high resolution and contain microstructures of cells, tissues. The research of micrographic image generation helps to address the challenges of scarcity, quality issues and lack of data diversity. Traditional image generation methods have difficulty in capturing subtle textures and diversity in images. In contrast diffusion models show strong advantages in these areas. Diffusion models are able to supplement data deficits by generating new images. Through synthesizing more images with different lesion types, the diffusion model helps to train deep learning models to improve automated analysis in pathology, cytology, and other fields. The application of diffusion models in microscopic image generation highlights their powerful generative capabilities, with significant advancements in both unconditional and conditional generation. The common conditional information in microscopic image generation are textual descriptions and image inputs. In Figure 9, it overviews diffusion-based approaches for generating microscopic and micro-alike images from three perspectives.

### 3.1 Unconditional image generation

**Figure 10:**
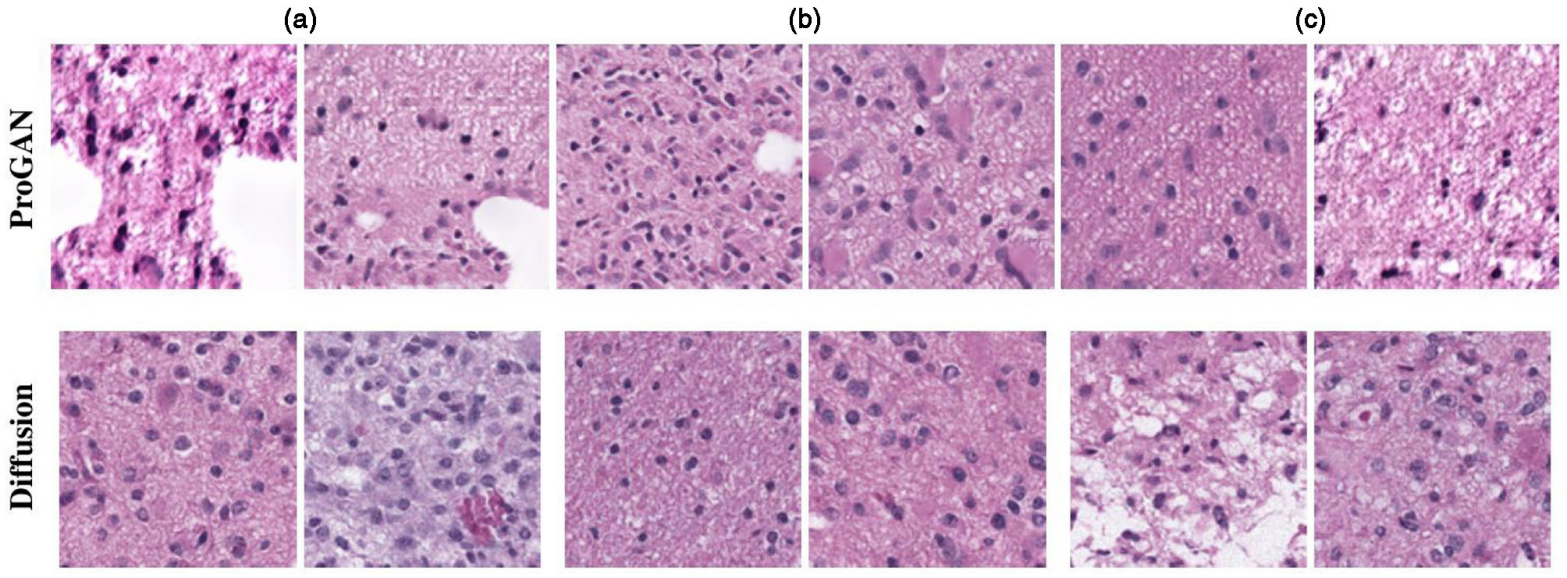
Results of histopathological image generation using the algorithm presented in (52). Reproduced with permission from “Selection of generated patches with diffusion and ProGAN models” by Puria Azadi Moghadam, Sanne Van Dalen, Karina C. Martin, Jochen Lennerz, Stephen Yip, Hossein Farahani and Ali Bashashati, licensed under arXiv.org perpetual, non-exclusive license 1.0.

Histopathological images are the gold standard for diagnosing many diseases, particularly cancer (51). For rare cancers, diffusion models can generate new images for examination. Several scholars have explored DDPM-based histopathological image generation. For instance, (52) introduced a DDPM-based method for genotype-guided generation of histopathological images. To enhance the model's focus on morphological patterns, input images are first converted to a uniform color domain using a color normalization module (53). Additionally, a perceptual priority weighting module (54) is employed, which emphasizes perceptual components of the image by applying higher weights to losses at earlier levels and lower weights at later stages. This approach achieves the generation of detailed and complex histopathological images, as shown in Figure 10, and demonstrates superiority over the ProGAN method.

In recent work (55), Medfusion, a conditional DDPM-based model, is introduced for medical image generation. The input image is first encoded by an autoencoder into an 8-times compressed latent space. In this latent space, the diffusion process of DDPM and U-Net denoising occurs, followed by decoding back into image space. In experiments comparing the quality of medical image reconstruction, the stable diffusion model's autoencoder with four channels produces artifacts that affect diagnosis. In contrast, Medfusion's 8-channel VAE more accurately reconstructs small structures, demonstrating its superior reconstruction capability. Additionally, in fundus image generation experiments on the AIROGS dataset, Medfusion outperforms StyleGAN-3 in terms of FID, KID, Precision, and Recall.

The cell cycle is a critical phase of the cell life cycle, and classifying its different stages is essential for understanding cellular biological processes and disease treatment. However, the mitotic phase is much shorter than interphase, leading to severe data imbalance and reduced classifier performance. To address this, (56) proposes using DDPM for mitotic phase data augmentation to balance the number of images at each phase. ResNet is then used to train a classifier on the original dataset, images generated by the WGAN-GP model, and the synthetic dataset. As a result, the M-phase classification metric, PPV, improved from 0.718 to 0.941, while the PPV for G1, G2, and S-phase data showed minimal difference from the original dataset. These results indicate that diffusion model-based data augmentation can effectively enhance classification model performance.

In (57), a modified DDIM is introduced to synthesize abnormal leukocyte images for classifier training. To improve model performance, the U-Net denoising module in DDIM is replaced by R2U-Net (58), which combines U-Net, RNN, and residual networks. The synthetic images are evaluated using FID, and the improved DDIM outperforms GAN-generated images in both quality and diversity.

### 3.2 Conditional image generation

In contrast to unconditional generation, conditional generation refers to the control of the output image by introducing additional conditional information. Conditional generation overcomes the randomness and uncontrollability of unconditional generation. The common types of conditions are labels, images, text, and others. This section focuses on text-condition and image-condition image generation.

#### 3.2.1 Text-condition image generation

In (59), a text-guided diffusion model GLIDE (60) (Guided Language to Image Diffusion for Generation and Editing) is proposed for the synthesis of histopathological images. It learns to associate similar text and image representations using CLIP, and create a link between these two modalities. While GLIDE without CLIP guidance results in higher quality images, however, CLIP guidance improves synthetic images. This model has surpassed the performance of another up-to-date generative model, DALL-E (61).

The work of (62) proposes an effective text-conditional latent diffusion model, the PathLDM model. Pathology reports were first summarized using GTP-3.5 (63) and leveraged as text conditions. PathLDM mainly consists of Variable Auto-Encoder (VAE), U-Net Denoiser and Text Encoder with additional refinements. The SSIM of the reconstructed images is greatly improved by using a VAE with a downsampling factor of 8. The application of fine-tuned U-net also further improves the generated results. What' more, To embed sufficiently medium-length text sequences, the OpenAI CLIP (64) is replaced with PLIP (65). As a consequence, the FID is improved from 48.14 to 7.64, and text-to-histopathology image generation is achieved on the TCGA-BRCA dataset (66).

The dermatological datasets available for labeled training are limited by issues such as privacy. Therefore, text-guided image generation for dermatological diseases based on stable diffusion model is proposed in (67). In order to separate out the low quality images generated, a generation pipeline is presented. The data generated by the stable diffusion model is first filtered out non-skin images via a binary EfficientNet classifier (68). A pre-trained ensemble model is then used to predict skin disease labels. Finally the correctly labeled images can be utilized to enhance the initial dataset. At last, the classifier is trained on real dataset, hybrid dataset, and synthetic dataset using convolutional neural network. The experimental results show an improvement in the accuracy of the classifiers trained on the hybrid dataset. Barriers to sharing labeled medical datasets are minimized without compromising classification performance.

Cervical cytology is a diagnostic method to determine cervical diseases by observing the morphological structure of cervical cells (69). Generating synthetic images with cervical cytological features using the fine-tuned stable diffusion model Dreambooth presented by (70). Thereby assisting the physician in the diagnosis and analysis of cervical disease. The model is fine-tuned using various regularization images, training images and step counting configurations (71). Dreambooth has two main processes: The low-resolution image is first created by a text-guided diffusion model. The super-resolution diffusion model is then used to generate higher quality images (72). Experimental results show that the fine-tuned stable diffusion model is capable of generating synthetic images with cervical cytological features.

#### 3.2.2 Image-condition generation

**Figure 11:**
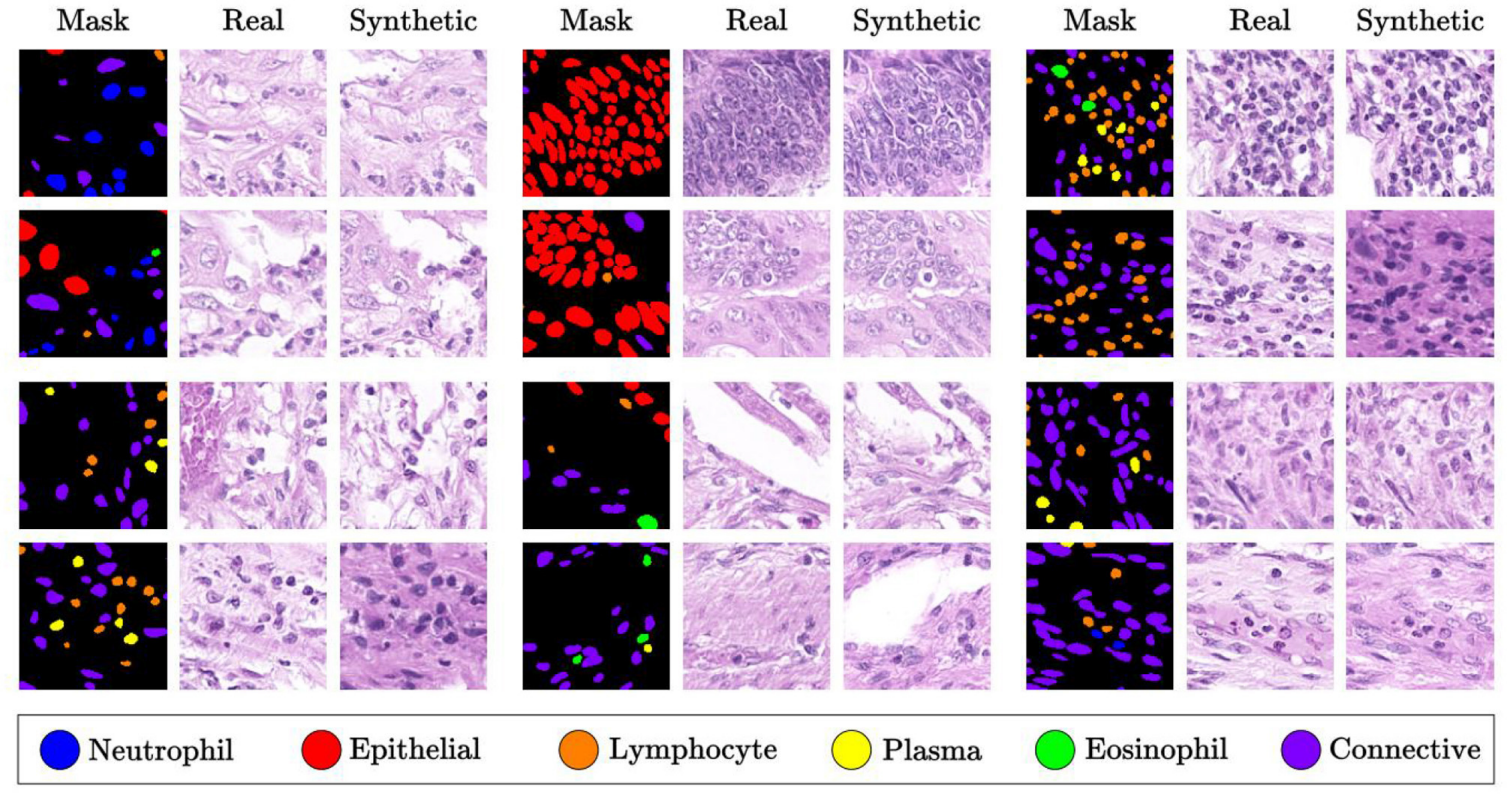
Synthetic images generated from masks for each type of nuclei, as described in (73). Reproduced with permission from “Qualitative Analysis” by Aman Shrivastava and P. Thomas Fletcher, licensed under CC BY 4.0.

The work of Shrivastava and Fletcher (73) introduces a method for generating synthetic images using DDPM, conditioned on segmentation masks of the nuclei. To ensure consistency in staining intensity and color distribution across tissue slice images, the staining is first normalized. During the denoising process, an improved U-Net architecture (74) is employed, embedding a multilayer spatial adaptive normalization operator in the decoder to retain and communicate semantic information throughout the generation process. Additionally, classifier-free guidance (75) is used to adjust the bootstrap weights of the conditional and unconditional generation distributions, enhancing image features. The synthetic images generated for different types of nuclei are shown in Figure 11. The Fréchet Inception Distance (FID) (76) and Inception Score (IS) achieved values of 15.7 and 2.7, respectively, outperforming GAN-based generative models and Morph-Diffusion models (52).

Human Epidermal growth factor Receptor 2 (HER2) is an important tumor marker that is closely associated with the development and prognosis of breast cancer (77). Due to the large quantitative differences between the different HER2 tumor subtypes, which results in a category imbalance in the training data. A generative model-based semantic conditional synthesis of HER2 data is presented in (78). To compensate for different HER2 tumor subtypes by modifying the label mask. HER2 histopathological images were synthesized using three different generative models, including GAN-generated (79), diffusion model (80) and diffusion model-inpainted. Furthermore, the synthetic data is added to the original data for tumor segmentation. when adding 100% synthetic images, the diffusion-generated images improved the Dice score (81) to 0.854. Outperforming the other two methods and attains the optimal indicators.

Aversa et al. (82) introduces a method for generating large-size, high-quality histopathological images called DiffInfinite. DiffInfinite uses a semi-supervised learning approach based on a joint Vector Quantised-Variational AutoEncoder(VQ-VAE) with a denoising diffusion implicit model. The image passes through the VQ-VAE encoder to the low-dimensional potential space, where forward diffusion is performed. Then, it is decoded back into pixel space (80). DiffInfinite utilizes a parallel random patch diffusion method to generate large size mask images. Experiments on the lung tissue datasets synthesize 2048 × 2048 px and 512 × 512 px images. The Improved Recall metrics (83) reach 0.98 and 0.94, respectively.

In cancer, abnormal expression of certain genes can promote the proliferation, invasion and metastasis of tumor cells, which leads to aberrant tissue morphology (84). To this regard, Carrillo-Perez et al. (85) proposes the RNA-CDM architecture, a gene expression-guided for multi-cancer RNA-to-image synthesis based cascade diffusion model. To generate RNA-Seq (86) embeddings, the expression of 17,655 genes are mapped to the latent space via the β-VAE architecture (87). The RNA-CDM architecture consists of two DDPMs. The first DDPM works with β-VAE architecture for multi-cancer synthesis of rna to 64 × 64 images. The other DDPM acts as a super-resolution model. A high resolution of 256 × 256 image is generated. Experiments on the dataset TCGA accurately synthesize 50k tiles with 10, 000 per cancer type. In addition, they used HoverNet (88) to detect different cell types in synthetic data. Demonstrated that the RNA-CDM architecture captures different morphological features of each cancer type.

Xu et al. (24) introduces visual transformers (ViTs) (89) into diffusion self-encoders (90), which are used to replace convolutional neural networks as semantic encoders. In the first stage, an input image is encoded into a semantic representation by the ViT. This representation is taken as the condition for the conditional DDIM to decode the noisy image. In the second stage, a latent DDIM is trained to learn the distribution of semantic representations of data. Then, feed it to the conditional DDIM along with randomly initialized noisy image to generate new histopathology samples. The experimental evaluation results FID on NCT - CRC, PCam, Chaoyang datasets are 12.14, 13.39, 36.18 respectively. Ultimately, the synthetic images are mixed with real images to train the classifier and the performance is improved.

Traditional staining of microscope images involves a physical process that is time-consuming. Virtual staining technology, which uses computational methods, can replace physical staining (91). It compared the performance of Diffusion Models and CycleGANs in virtual staining, translating slice-free microscope images (SFM) to H&E images. The Dual Diffusion Implicit Bridges (DDIB) model, a variant of the diffusion model, was used to achieve this [135]. DDIB combines implicit generation and denoising diffusion techniques to improve generation efficiency while maintaining high-quality output. However, the translation results on the MUSE-to-H&E and FIBI-to-H&E datasets revealed that DDIB suffers from feature omission, and CycleGAN performs better in retaining the semantic features of the original image. Additionally, DDIB underperforms compared to CycleGAN in external critic accuracy and FID metrics.

### 3.3 Summary

From the above literature summary, it is clear that diffusion models have a wide range of promising applications in image generation. We have listed the datasets used for each model. Whether histopathological images, cellular images or skin cancer images, high quality images are produced. In addition, The diffusion model outperforms the GAN-based model in terms of quality of the generated microscopic images, image diversity, and model stability.

## 4 Application of image segmentation

**Figure 12:**
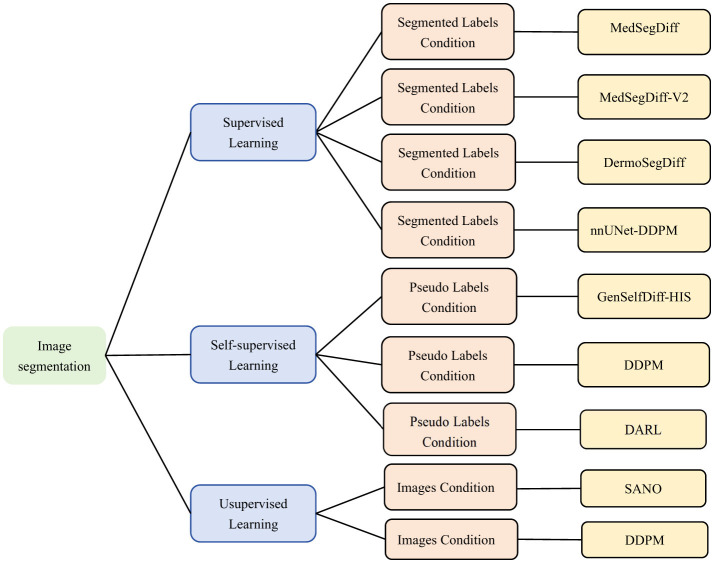
Classification of microscopic image segmentation based on diffusion model. Supervised Learning (93, 96, 98), (100), Self-supervised Learning (104, 108, 110), Unsupervised Learning (112, 113). We use the following abbreviations in the architecture column: MedSegDiff, Medical Image Segmentation with Diffusion Probabilistic Model; MedSegDiff-V2, Diffusion-based Medical Image Segmentation with Transformer; DermoSegDiff, A Boundary-aware Segmentation Diffusion Model; GenSelfDiff-HIS, Generative Self-Supervision Diffusion Model; DARL, Diffusion Adversarial Representation Learning.

Image generation not only provides training data, but can also be directly combined with segmentation models to improve segmentation accuracy (92). Image segmentation requires a lot of labeled data. While labeling high quality medical images, microscopic images is very expensive and time consuming. The images generated by the diffusion model can be used with real data to train image segmentation models. In addition, diffusion models can be combined with self-supervised learning or weakly supervised learning. Thus, effective image segmentation can be performed without fully labeled data. The reverse denoising process of the diffusion model can also be used as an unsupervised learning method. Extracting potential structural information from unlabeled images and providing assistance for segmentation tasks. The combination of self-supervised learning methods and diffusion models can significantly improve the effectiveness of segmentation models in the case of scarce labeled data. As shown in the Figure 12, this section will introduce the application of diffusion model on image segmentation from supervised, self-supervised, and unsupervised learning.

Moreover, standardized segmentation algorithms can help reduce subjective factors and human errors in diagnosis. This section covers image segmentation algorithms based on diffusion models.

### 4.1 Diffusion Model with supervised learning

Microscopic image segmentation is a complex yet essential task. In (93), a DDPM-based segmentation model, MedSegDiff, is introduced for optical cup segmentation. MedSegDiff employs an improved ResUnet (94) in the denoising process and introduces dynamic conditional coding to better leverage medical image features. At each time step, the condition information is fused with the current state using an attention-like mechanism, allowing the conditional information to have varying effects at different time steps. To address high-frequency noise generated during this fusion, the Feature Frequency Parser (FF-Parser) is proposed, which removes high-frequency noise by modulating the spectrum in the frequency domain using 2D FFT. Segmentation results on the REFUGE-2 dataset (95) show that MedSegDiff outperforms most baseline models.

The main structure of MedSegDiff is based on the UNet network (93). Given the recent success of visual transformers in medical image segmentation, the authors further improved MedSegDiff by proposing the MedSegDiff-V2 model, which combines a transformer-based UNet with DDPM (96). To reduce diffusion variance, the model uses two conditioning methods: anchoring condition, which integrates segmentation features into the diffusion model encoder, and Spectrum Space Transformer (SS-Former), which learns the interaction between noise and semantic features. The Dice coefficient and IoU for optical cup segmentation in fundus images reach 87.9 and 80.3, respectively, indicating that MedSegDiff-V2 outperforms both MedSegDiff and previous state-of-the-art methods.

**Figure 13:**
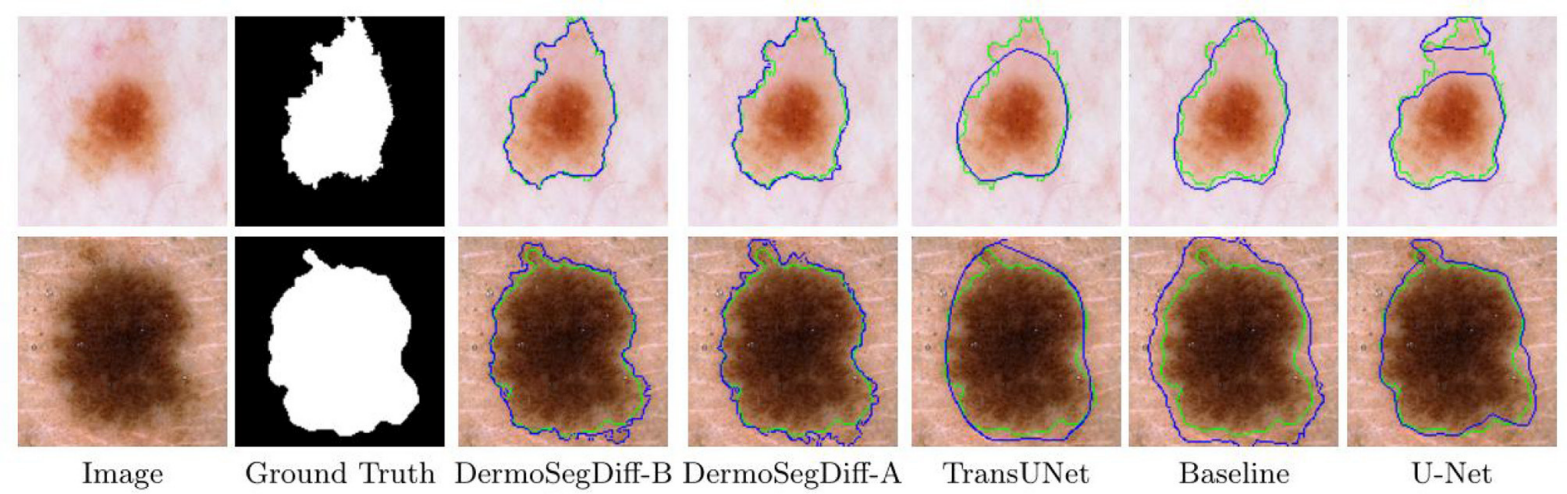
Visual comparisons of different methods on the ISIC 2018 skin lesion dataset. Reproduced with permission from “Visual comparisons of different methods on the ISIC 2018 skin lesion dataset. Ground truth boundaries are shown in green, and predicted boundaries are shown in blue” by Afshin Bozorgpour, Yousef Sadegheih, Amirhossein Kazerouni, Reza Azad and Dorit Merhof, licensed under CC BY 4.0.

Skin lesions often have complex shapes and irregular borders, making accurate boundary detection crucial for precise segmentation (97). To enhance segmentation accuracy and precision, a boundary-aware diffusion model, DermoSegDiff, is proposed (98). DermoSegDiff introduces a boundary-aware module into the diffusion process, where the lesion area's boundary information is detected in real-time using a distance transform function (99). This boundary information guides pixel adjustments during the generation process to ensure clear and accurate boundaries. Additionally, a loss function incorporating boundary information is designed to focus on both region segmentation accuracy and boundary clarity. An improved denoising network architecture is also presented to accelerate convergence. As shown in Figure 13, DermoSegDiff effectively captures complex boundaries compared to the baseline state-of-the-art methods.

Segmentation of histopathological whole-slide images presents challenges due to data scarcity and annotation difficulties. (100) proposed a cascade diffusion model conditional on segmentation label masks. In the first stage, an unconditional diffusion model generates synthetic images, which are then segmented using a UNet model to obtain segmentation masks. In the second stage, a conditional diffusion model uses the synthetic image segmentation mask and noise image from the previous stage to generate the final synthetic image. This image is used to expand the dataset, and performance is evaluated using nnUNet (101) segmentation. The results show that the conditional diffusion model outperforms the unconditional model in image segmentation, and segmentation models fine-tuned with synthetic images show significant performance improvements.

### 4.2 Diffusion model with self-supervised learning

In medical diagnosis, pathology image segmentation is critical. Traditional segmentation methods (102, 103) often rely on fully supervised learning, which requires large labeled datasets. Self-supervised learning offers advantages in reducing labeling costs, and DDPM typically outperforms GANs and VAEs in image quality. Therefore, Purma et al. (104) combines DDPM with self-supervised learning for pre-training on unlabeled data, followed by fine-tuning with a UNet for histopathological image segmentation. To address unbalanced data, multiple loss functions, including structural similarity (SS) loss (105) and focal loss (FL), are introduced. The results show that GenSelfDiff-HIS significantly improves segmentation performance compared to other self-supervised and supervised learning methods.

Cell instance segmentation is a key task in biomedical image analysis. The aim is to accurately segment overlapping or touching cells into separate instances. The symmetry issue is a major challenge because models have difficulty distinguishing and segmenting similarly shaped cell (106, 107). Hereby, an approach to cell instance segmentation based on the diffusion model is proposed (108). With the introduction of Spontaneous Symmetry Breaking(SSBs) in the diffusion process, the model is capable of better distinguishing and segmenting symmetric cell instances. The model parameters are optimized by combining traditional segmentation losses (e.g., cross-entropy loss, Dice loss) and losses specific to symmetry breaking. The experimental results on fluorescent cell data (109) validate the effectiveness and superiority of the proposed method in dealing with overlapping cell segmentation.

In addition to the studies mentioned earlier, Kim et al. (110) proposed Diffusion Adversarial Representation Learning (DARL), which combines a diffusion model with a self-supervised learning method. The DARL model comprises diffusion and generation modules along with a discriminator, incorporating a switchable version of SPADE (79) in the generation module. The model's training process involves two main paths: the diffusion path and the adversarial path. The diffusion path focuses on learning global and local features of an image, providing a rich feature representation for subsequent segmentation tasks. The adversarial path, using PatchGAN (111), ensures that the model generates realistic segmentation results. The DARL model has been applied to segment the external retinal image dataset, where it outperforms existing unsupervised and self-supervised methods.

### 4.3 Diffusion model with unsupervised learning

Traditional skin lesion detection requires large amounts of annotated data, which is often time-consuming, costly, and prone to subjective differences that can affect the model's generalization ability. The SANO model addresses this by combining a diffusion model with a score-based approach to improve the detection and localization accuracy of skin lesions using unsupervised learning (112). This approach reduces the time and cost associated with data labeling and achieves better results in hand eczema detection compared to other unsupervised methods.

Shao et al. (113) introduced a semi-supervised cell nucleus segmentation framework based on unsupervised pre-training. This framework consists of three main steps: first, unsupervised pre-training on a large number of unlabeled images using a latent diffusion model; second, aggregation of feature mappings from different denoising blocks using a transformer-based decoder (114), with pre-trained diffusion models serving as feature extractors to generate pseudo-annotations and extend the labeled dataset; and finally, integrating predictions from multiple models using collaborative learning (115) to further enhance segmentation performance. The experiments demonstrated significant improvements in cell nucleus segmentation compared to semi-supervised and supervised baselines.

### 4.4 Summary

The section above outlines the application of diffusion model-based image segmentation in microscopic and micro-alike images, covering relevant references, segmentation models, datasets, and other key information. Most DDPM-based diffusion models utilize self-supervised or unsupervised training to minimize reliance on manual labeling, thereby reducing the risk of overfitting associated with small labeled datasets. Moreover, the results consistently show significant improvements compared to traditional methods.

## 5 The diffusion model for other applications

**Figure 14:**
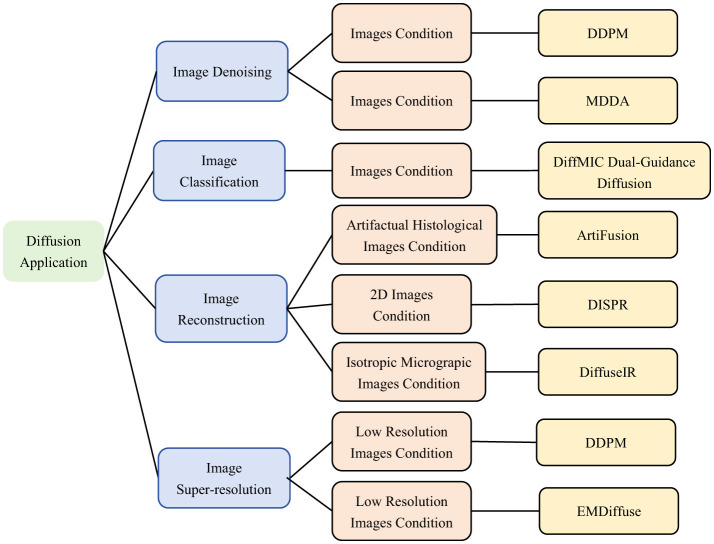
Classification of Diffusion based model Applications.Including image Denoising (26, 119), image Classification (121), image Reconstruction (122, 125, 129), image Super-Resolution (131, 133). We use the following abbreviations in the architecture column: MDDA, Multiscale Diffusive and Denoising Aggregation Mechanism; DiffMIC, Dual-Guidance Mechanism; ArtiFusion, Artifact Restoration with Diffusion Probabilistic Models; DISPR, Diffusion-Based Image Shape Prediction and Reconstruction; DiffuseIR, Diffusion Models for Isotropic Reconstruction of 3D Microscopic Images; EMDiffuse, Expectation-Maximization Diffusion Model.

The successful application of the diffusion model as a powerful generative model for image generation and segmentation lays the foundation for other tasks. Its main strengths include denoising, generating high-quality details, and data enhancement. These advantages can be useful in several application scenarios, in fields such as image translation, self-supervised learning, target detection, and medical image analysis. As the diffusion model continues to evolve, it will demonstrate its powerful generative capabilities and versatility in more applications. As illustrated in Figure 14, it will present other application areas of diffusion model on microscopic images.

### 5.1 Image denoising

Optical Coherence Tomography (OCT) is a high-resolution imaging technique prone to optical noise and interference patterns, such as speckle noise. To address this issue, an unsupervised denoising approach using DDPM is proposed in (26). During pre-processing, a self-fusion method (116) is applied to obtain a clearer input image. Subsequently, DDPM gradually denoises the image while controlling the denoising step with a time signal to prevent excessive smoothing. This method outperforms the Pseudo Modal Fusion Network (PMFN) (117) in both noise suppression and detail retention.

Adversarial samples can mislead deep learning models by making small perturbations to the original image, causing classification errors. This poses significant safety concerns, particularly in sensitive applications like dermatological testing (118). To counteract this, Wang et al. (119) introduces the Multi-scale Diffusion and Denoising Aggregation (MDDA) mechanism, which effectively defends against and reverses adversarial samples in skin cancer images. The process involves multi-scale image processing to preserve structural features, followed by adversarial noise removal via a diffusion model. The denoised image is then fused with images from neighboring scales through an aggregation process. Experimental results demonstrate that MDDA offers strong defense against various adversarial attacks, outperforming other defense methods.

### 5.2 Image classification

**Figure 15:**
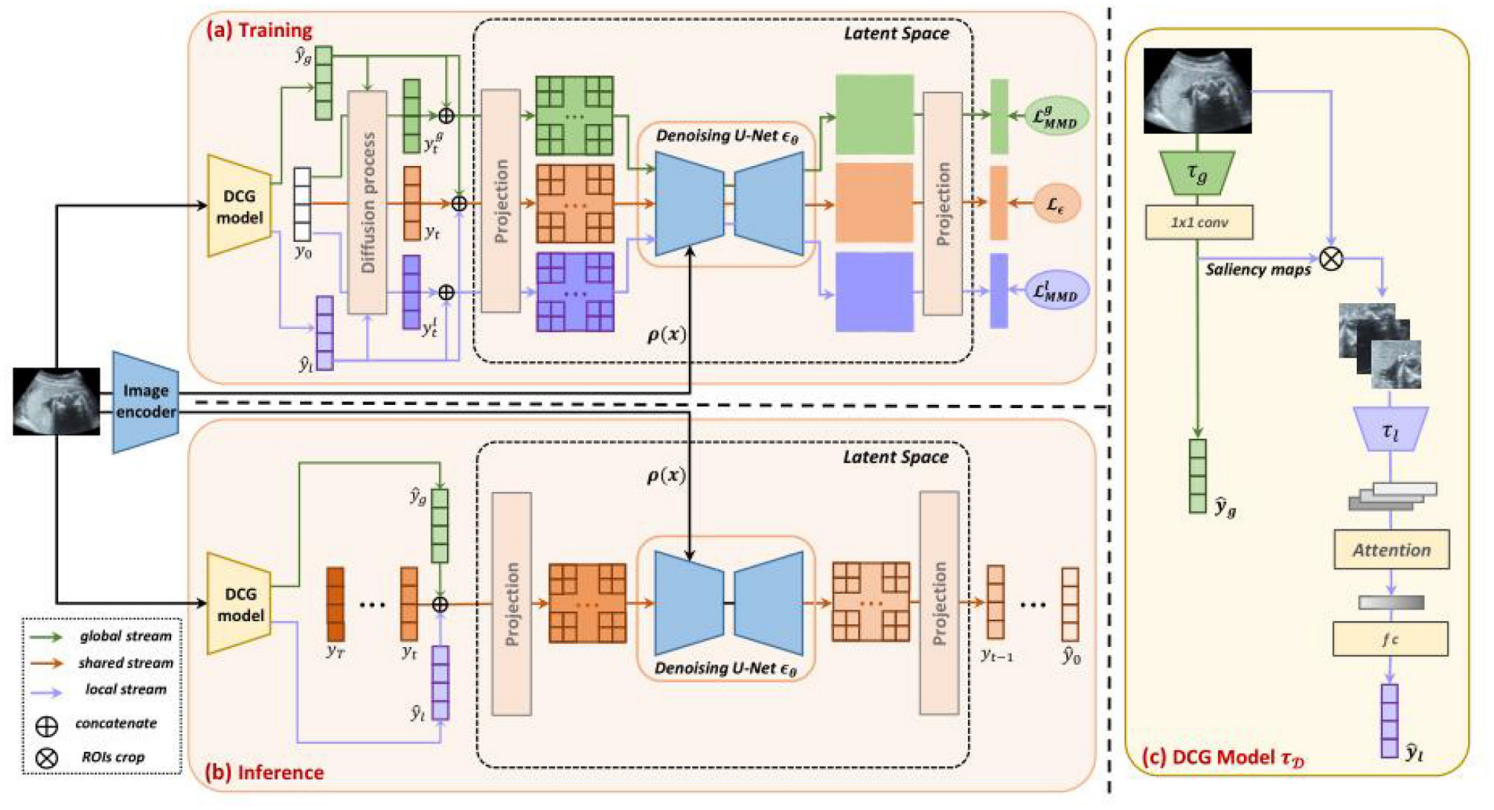
Dual-guidance mechanism of DiffMIC. Reproduced with permission from “Overview of our DiffMIC framework” by Yijun Yang, Huazhu Fu, Angelica I. Aviles-Rivero, Carola-Bibiane Schönlieb and Lei Zhu, licensed under CC BY 4.0.

With the increasing use of deep learning in medical image analysis, accurate classification of medical images has become crucial (120). Traditional classification methods face challenges, such as variations in image quality and limited labeled data, especially with complex medical images. To address these challenges, Yang et al. (121) introduced the Dual-Guidance Diffusion Network for Medical Image Classification (DiffMIC). This approach enhances classification accuracy and robustness through a Dual-Guidance (DG) mechanism. As illustrated in Figure 15, DiffMIC leverages two types of guidance: label-based supervision and diffusion information from unlabeled data. The core of DiffMIC is a two-branch network architecture. The first branch is a conventional CNN that extracts image features and performs classification, while the second branch is a diffusion network that learns implicit structural information from unlabeled data. By fusing information from both branches, DiffMIC achieves a deeper understanding and more accurate classification of medical images. Additionally, DiffMIC introduces Maximum Mean Discrepancy (MMD) regularization to minimize differences between the feature distributions of labeled and unlabeled data. Experimental results demonstrate that DiffMIC outperforms existing mainstream methods, showing superior accuracy and F1 scores on publicly available micro-alike images.

### 5.3 Image reconstruction

Histological images often suffer from various artifacts, such as uneven staining, noise, and refraction artifacts, which can impede pathologists' analysis and diagnosis. Traditional image restoration methods typically rely on labeled training data, which is costly and difficult to obtain. To address this, He et al. (122) proposed ArtiFusion, an unsupervised diffusion-based model for histological artifact restoration. ArtiFusion generates images with artifacts through a diffusion process and then restores them via a reverse process, relying solely on artifact-free images. The model replaces the U-Net network with the Swan-Transformer denoising architecture (123), and introduces a time token within the architecture, enabling the model to accurately recognize and process images with varying noise levels. Compared to CycleGAN (124), ArtiFusion demonstrates superior performance across several metrics, including SSIM, PSNR, FSIM, and SRE.

Microscope imaging techniques are widely used in the biomedical field, but they typically provide only two-dimensional images of samples. Reconstructing the three-dimensional (3D) structure of biological samples often requires multi-angle imaging or specialized 3D microscopic techniques, which can be complex, expensive, and time-consuming. To address this, Waibel et al. (125) proposed DISPR, a diffusion-based shape prediction model that reconstructs 3D cell shapes from 2D microscope images. By training five independent DISPR models simultaneously, the stochastic nature of the diffusion model allows for the prediction of five different 3D cell images per 2D input. DISPR achieves a lower relative volume error compared to SHAPR (126) and SHAPR with topological loss (127), and also outperforms other models in surface area error, surface roughness error, and relative surface curvature error.

In medical imaging, high-resolution 3D images often face anisotropic resolution challenges, where cross-sectional images have higher resolution than vertical images (128). To address this, Pan et al. (129) introduced DiffuseIR, an unsupervised diffusion model designed to improve isotropic super-resolution reconstruction. DiffuseIR incorporates Sparse Spatial Condition Sampling (SSCS) during the inverse diffusion process to guide the denoising process by exploiting spatial structure conditions, thereby improving accuracy and reducing blurring and distortion due to anisotropy. To address texture incoherence, a Refine-in-loop Strategy is integrated into SSCS, allowing the model to correct and reduce errors in each reconstruction cycle based on the previous round's results. Compared to conventional methods, such as bicubic interpolation, and supervised super-resolution methods, DiffuseIR produces 3D images with superior quality.

### 5.4 Image super-resolution

Super-resolution microimaging enables researchers to observe structures smaller than the resolution limits of conventional microscopes (130). Saguy et al. (131) proposed a diffusion model-based method for generating high-quality super-resolution microscopic images. To ensure the synthetic images retain the features and structure of the training data, cross-correlation scores between each generated image and augmented patches from the training set are calculated. The image with the highest cross-correlation score is selected for evaluation. Additionally, Content-Aware Recovery (CARE) is trained using diffusion-based and mathematical model-based microtubule images (132), validating the superior reconstruction quality of the diffusion-based model. Importantly, this diffusion-based super-resolution method generalizes across different types of image data, a feat not possible with traditional mathematical models.

**Figure 16:**
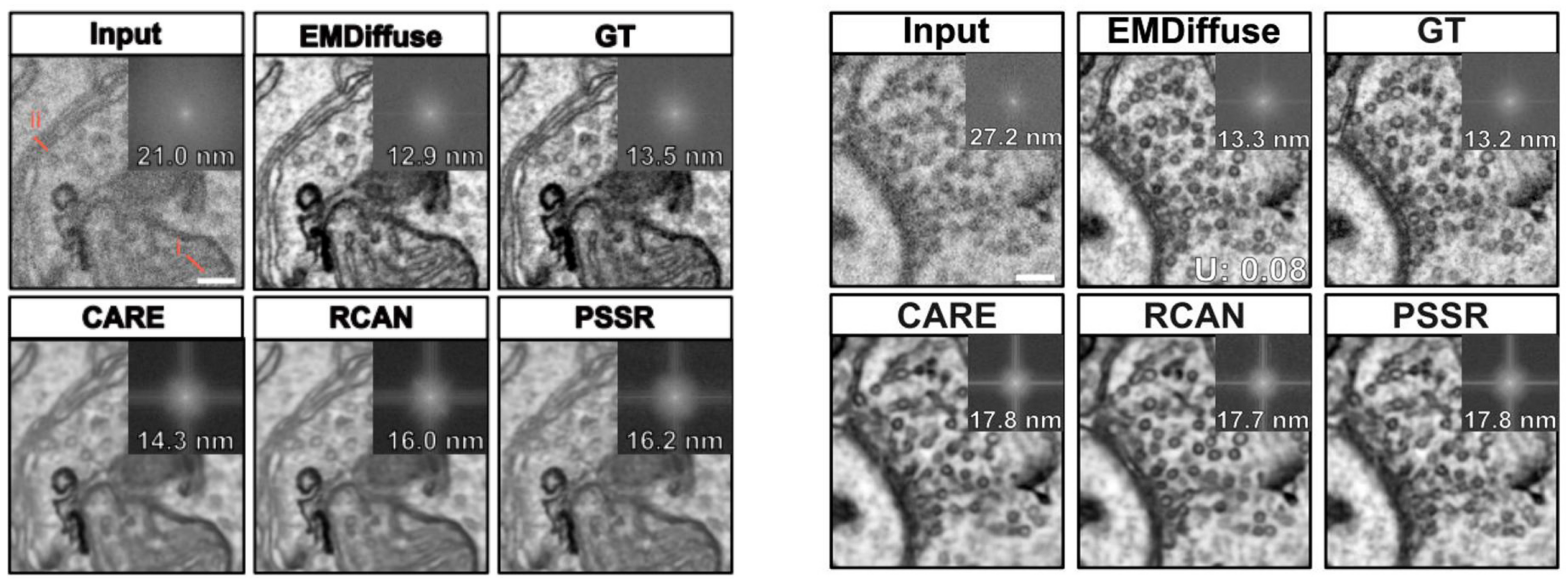
**Left**: Comparison of the denoising ability of EMDiffus-n with CARE, PSSR and RCAN. **Right**: Comparison of EMDiffus-r super-resolution with CARE, PSSR and RCAN. Top-right of each panel is the Fourier power spectrum. Adapted with permission from “EMDiffuse exhibits excellent denoising capability and generates images with high-resolution ultrastructural details” by Chixiang Lu, Kai Chen, Heng Qiu, Xiaojun Chen, Gu Chen, Xiaojuan Qi and Haibo Jiang licensed under CC BY 4.0.

Electron microscope (EM) imaging often encounters noise, particularly in low-dose imaging, and requires overcoming numerous technical challenges to obtain high-quality ultra-structural images. To address this, Lu et al. (133) introduced EMDiffuse, an innovative deep learning method based on diffusion models, aimed at enhancing the quality of EM images through denoising and image enhancement. In the preprocessing stage, ORB (134) and optical flow estimation (135) are used to align the original image with the real reference image. EMDiffuse employs a U-Net architecture with a global attention layer between the encoder and decoder to capture global information and dependencies (28). EMDiffuse excels in tasks such as image denoising, super-resolution, and reconstruction for microscopic images. As shown in Figure 16, EMDiffuse-n preserves the resolution and ultra-structure of microscopic images during denoising, outperforming other algorithms like CARE28, RCAN38, and PSSR23 in distinguishing organelles such as mitochondria and cell vesicles. EMDiffuse-r successfully improves image resolution from 6 nm to 3 nm in super-resolution tasks. Moreover, EMDiffuse demonstrates versatility by performing well across different datasets, such as mouse cerebral cortex, liver, heart, and bone marrow. The model also supports 3D microscopic image reconstruction, addressing anisotropic resolution issues. VEMdiffusion-i achieves isotropic resolution in vEM imaging by generating an intermediate layer between two anisotropic volumes, increasing imaging speed by a factor of 5. VEMdiffusion-a further enables isotropic volume reconstruction using only anisotropic training data, with VEMdiffusion-i trained along the z-axis and VEMdiffusion-a along the y-axis of the isotropic volume.

### 5.5 Summary

In summary, we conclude the applications of diffusion models in the different fields, including denoising, reconstruction, super-resolution, classification, and translation of images. The flexibility and powerful generative capabilities of diffusion model have shown its potential for a wide range of applications. Each task is an improvement of the base diffusion model with the use of existing technology. Moreover, remarkable results have been achieved on both microscopic and microscopic-like images. Nevertheless, the diffusion model fails to outperform the Gan-based model in terms of image translation. Hence, the application of diffusion model in this area requires further research.

## 6 Method analysis and potential

Diffusion Models have performed well in the field of image generation and analysis. However, there are still some limitations and challenges. Following are the major limitations of Diffusion Models and their impact in applications: High computational cost of training: Diffusion models require learning complex probability distributions over multiple steps of diffusion and inverse diffusion. Each step requires training a large number of parameters. This leads to longer training times and higher hardware requirements. Especially when generating high resolution images, it needs strong GPU/TPU support. It is not friendly enough for small research institutions or users with limited resources, restricting its popularity and application.

The sampling process is slow: The generation of images requires a long single generation time, especially in high-resolution tasks. Although some of the methods (e.g., DDIM, FastDPM) try to speed up sampling, fast sampling usually reduces the generation quality. This makes it difficult to apply diffusion models to real-time tasks or scenarios where results need to be generated quickly.

Highly data-dependent: Diffusion models require large, high-quality training data to learn complex distributions. Insufficient samples may result in degradation of the quality of the generated images and also Out-of-Distribution (OOD) errors. In scarce data scenarios, such as the microscopic images studied in this paper, the model performance may be less than optimal.

This section discusses the advantages and limitations of the DDPM, DDIM, and SANO models, respectively.

### 6.1 Analysis of DDPM methods

As highlighted in the review, DDPM is one of the most widely used diffusion models, particularly in micrography. DDPM has shown great potential in image generation, segmentation, and super-resolution. Its core idea is to gradually refine the generated image through multiple denoising steps, enabling better recovery of details and high-frequency information. DDPM progressively reduces noise during the inversion process, optimizing generation quality at each step, resulting in detail-rich images. For instance, (55) used DDPM to synthesize histopathological images of colon cancer and fundus images, achieving FIDs of 30.03 and 11.63, respectively. Similarly, DDPM synthesized cells in the mitotic M phase with a PPV of 0.941 (57). The stability of the denoising process, where each step builds on the previous one, contributes to a more steady training process. Additionally, DDPM avoids the instability associated with adversarial training by relying solely on maximum likelihood estimation. Furthermore, DDPM can train on unlabeled data, making it suitable for large-scale unsupervised learning.

#### 6.1.1 Limitations

Despite its advantages, DDPM has some limitations. Both the forward and reverse processes involve multiple iterative steps, each requiring complex calculations, which demand significant computational resources and time (136). Additionally, DDPMs typically rely on deep CNNs (e.g., UNet) for noise prediction and denoising, which contain numerous parameters and computational operations (137). These networks have complex structures, leading to long training and inference times, making real-time applications challenging. Moreover, training high-quality generative models generally requires large-scale datasets.

### 6.2 Analysis of DDIM methods

Unlike DDPM, DDIM is capable of generating high-quality images in fewer steps, significantly reducing generation time. The key innovation of DDIM is the introduction of deterministic mapping, which allows for more efficient sampling without relying on random noise during the process. This deterministic approach enables more accurate restoration of image details, improving the quality and stability of the generated images.

For example, (82) combined a VQVAE encoder with DDIM, achieving image evaluation indices of IR and IP at 0.94 and 0.7, respectively. This method not only enhances sampling speed but also maintains high image quality. Additionally, DDIM was used with Vision Transformers (ViT) to synthesize histopathological images of colorectal cancer and breast lymphoid sections (24), resulting in FIDs of 13.39 and 36.18, respectively, indicating a close match to the real data distribution.

#### 6.2.1 Limitations

Although DDIM reduces the number of generation steps, each step remains computationally intensive, requiring substantial computational resources. The deterministic denoising process introduces new hyperparameters, which may necessitate extensive experimentation and debugging to optimize (138). Additionally, DDIM's performance is highly dependent on the distribution of the training data, potentially limiting its generalization ability in scenarios with significant variations in data distribution. The deterministic nature of DDIM can also limit the diversity of generated results compared to stochastic processes (139). Despite the increase in generation speed, further optimization is required to meet the demands of real-time applications. Scalability remains a challenge for processing large-scale data and high-resolution images, highlighting the need for continued research into optimization strategies.

### 6.3 Analysis of SDEs methods

Compared to DDPM and DDIM, Stochastic Differential Equations (SDEs) produce higher quality and more detailed images by optimizing the noise addition and removal processes. In terms of generation efficiency, SDEs improve sampling steps, significantly reducing computation and increasing efficiency. SDEs also enhance stability and robustness during training, minimizing issues such as pattern collapse and gradient vanishing. Noise optimization further improves the model's robustness to input noise, generating more consistent results. For instance, a Dice score of 0.358 was achieved in hand eczema detection using the SDEs model combined with unsupervised learning (112).

#### 6.3.1 Limitations

However, SDEs still demand substantial computing resources and have high memory requirements. The performance of the generated models is heavily dependent on the quality and diversity of the training data. In cases of insufficient or low-quality data, the effectiveness of SDEs may be compromised.

## 7 Conclusion and future work

This paper summarizes the methodologies of diffusion model-based analysis for micrographic and micro-alike images. We presented three diffusion models–DDPM, DDIM, and SDEs–and reviewed their applications in microscopic image generation, segmentation, denoising, classification, super-resolution, and reconstruction. Additionally, we compared the strengths and weaknesses of these models in terms of output image quality, generation efficiency, and model stability and robustness.

While diffusion models are a novel and promising generative approach, challenges such as multi-step training and sensitivity to noise still hinder their broader application in microscopic image analysis. The issues discussed in chapter six remain unresolved. Recent advancements in optimization algorithms and parallel computing techniques offer potential solutions to improve the efficiency of diffusion models, particularly in achieving real-time microscopic image generation (15). We discuss potential future research directions for diffusion model as follows.

### 7.1 Acceleration algorithm

The complex computational process is a significant challenge for diffusion models compared to other generative models (140, 141). Pruning and quantization are popular model compression techniques designed to reduce computational complexity and memory requirements (142, 143). The main focus of improvements in diffusion models is to develop more efficient training and generation algorithms. By applying pruning, quantization, and other techniques, the model can be compressed to become more lightweight and suitable for practical applications.

### 7.2 Adaptive noise scheduling

In micrography, which involves studying and analyzing tiny structures, high-quality images are essential. Optimizing the noise addition and removal process in diffusion models can significantly enhance the quality of generated images. Adaptive Noise Scheduling dynamically adjusts the noise level based on the quality of the generated image and feedback from the model (144). A feedback mechanism calculates quality metrics (e.g., reconstruction error, perceptual loss) and adjusts the noise size for subsequent steps, allowing for more flexible control of noise. This approach prevents excessive or insufficient noise, improving detail and overall image quality.

### 7.3 Improving the generalization of models

The generalization ability of a model is crucial for its performance in real-world applications. Enhancing generalization reduces overfitting and improves accuracy on unseen data. This can be achieved through several strategies: expanding the training data using various data augmentation techniques, applying transfer learning to utilize pre-trained models for new tasks, and training the model on multiple related tasks simultaneously to boost generalization and generation quality.
